# Angiotensin-Converting Enzyme Insertion/Deletion Polymorphism Contributes High Risk for Chronic Kidney Disease in Asian Male with Hypertension–A Meta-Regression Analysis of 98 Observational Studies

**DOI:** 10.1371/journal.pone.0087604

**Published:** 2014-01-31

**Authors:** Chin Lin, Hsin-Yi Yang, Chia-Chao Wu, Herng-Sheng Lee, Yuh-Feng Lin, Kuo-Cheng Lu, Chi-Ming Chu, Fu-Huang Lin, Sen-Yeong Kao, Sui-Lung Su

**Affiliations:** 1 Graduate Institute of Life Sciences, National Defense Medical Center, Taipei, Taiwan, ROC; 2 School of Public Health, National Defense Medical Center, Taipei, Taiwan, ROC; 3 Division of Nephrology, Department of Medicine, Tri-Service General Hospital, National Defense Medical Center, Taipei, Taiwan, ROC; 4 Division of Pathology, Tri-Service General Hospital, National Defense Medical Center, Taipei, Taiwan, ROC; 5 Division of Nephrology, Department of Medicine, Shuang Ho Hospital, Graduate Institute of Clinical Medicine, Taipei Medical University, New Taipei City, Taiwan, ROC; 6 Division of Nephrology, Department of Medicine, Cardinal Tien Hospital, School of Medicine, Fu Jen Catholic University, New Taipei City, Taiwan, ROC; Max-Delbrück Center for Molecular Medicine (MDC), Germany

## Abstract

**Background:**

Associations between angiotensin-converting enzyme (ACE) gene insertion/deletion (I/D) polymorphisms and chronic kidney disease (CKD) have been extensively studied, with most studies reporting that individuals with the D allele have a higher risk. Although some factors, such as ethnicity, may moderate the association between ACE I/D polymorphisms and CKD risk, gender-dependent effects on the CKD risk remain controversial.

**Objectives:**

This study investigated the gender-dependent effects of ACE I/D polymorphisms on CKD risk.

**Data sources:**

PubMed, the Cochrane library, and EMBASE were searched for studies published before January 2013.

**Study eligibility criteria, participants, and interventions:**

Cross-sectional surveys and case–control studies analyzing ACE I/D polymorphisms and CKD were included. They were required to match the following criteria: age >18 years, absence of rare diseases, and Asian or Caucasian ethnicity.

**Study appraisal and synthesis methods:**

The effect of carrying the D allele on CKD risk was assessed by meta-analysis and meta-regression using random-effects models.

**Results:**

Ethnicity [odds ratio (OR): 1.24; 95% confidence interval (CI): 1.08–1.42] and hypertension (OR: 1.55; 95% CI: 1.04–2.32) had significant moderate effects on the association between ACE I/D polymorphisms and CKD risk, but they were not significant in the diabetic nephropathy subgroup. Males had higher OR for the association between ACE I/D polymorphisms and CKD risk than females in Asians but not Caucasians, regardless of adjustment for hypertension (*p*<0.05). In subgroup analyses, this result was significant in the nondiabetic nephropathy group. Compared with the I allele, the D allele had the highest risk (OR: 3.75; 95% CI: 1.84–7.65) for CKD in hypertensive Asian males.

**Conclusions and implications of key findings:**

The ACE I/D polymorphisms may incur the highest risk for increasing CKD in hypertensive Asian males.

## Introduction

The prevalence of chronic kidney disease (CKD) is approximately 10% in several countries [1]–[3]. CKD patients have high risk for cardiovascular disease and death [4]. Genetic factors, including ethnicity [5] and family history of disease [6], , play a key role in CKD pathogenesis. Thus, it is desirable to identify candidate genes and evaluate their effects.

The renin–angiotensin system (RAS) regulates blood pressure and electrolyte balance [8]. Owing to the key role of angiotensin-converting enzyme (ACE) in RAS, ACE polymorphisms have frequently been investigated. One of the most important ACE polymorphisms is a 287-bp insertion/deletion in intron 16 (ACE I/D), and a previous study revealed a significant effect of this polymorphism on ACE gene expression [9]. Most studies have found that carriers of the D allele had a higher risk of CKD or end-stage renal disease (ESRD) than those of the I allele [10]–[12].

Because previous studies have found that gender and the DD genotype had an additive effect on blood ACE levels [13], we hypothesized that gender differences affect the relative risk of ACE I/D polymorphisms for CKD. Numerous studies of CKD or ESRD have reported appreciable, but not significant, gender-dependent effects of ACE I/D polymorphisms [14]–[19], but the populations used in these studies were of different ethnicities. Studies of Caucasian subjects have indicated additive effects of the D allele in females [14]–[16], but studies of Asian subjects have shown different results [17]–[19]. Although many previous meta-analysis studies investigating ACE I/D polymorphisms and CKD have been reported, but no studies have considered moderate effects of gender in our knowledge [10]–[12], [20]–[22]. This study focused on general population without genetic abnormality or rare disorder, and we wanted to compare the risk of CKD in people with major allele (I allele) or minor allele (D allele) on ACE I/D. In addition, gender-dependent effects of ACE I/D polymorphisms on CKD risk was investigated.

## Methods

### Search Methods and Criteria for Considering Studies

The PRISMA 2009 Checklist was reported in Table S1. This study focused on the general population without genetic predisposing factors, and aimed to compare CKD risk between individuals carrying the major (I) and minor (D) alleles of ACE I/D. To identify relevant studies, English-language articles in MEDLINE, Cochrane Library and EMBASE were searched using relevant text words and medical subject headings that included all spellings of ACE I/D and CKD (the detailed search strategy is shown in Table S2). All articles published from the dates of inception of these medical databases to January 2013 were included. We manually scanned the reference lists of identified trials and review articles to avoid missing any other relevant studies [10]–[12], [20]–[22].

All related studies assessing the association between ACE I/D polymorphisms and CKD risk were considered for inclusion. The criteria for inclusion of a study were as follows: (1) cross-sectional surveys or case–control studies; (2) CKD defined according to the National Kidney Foundation: kidney damage by clinical diagnosis or a glomerular filtration rate <60 ml/min/1.73 m^2^; (3) presence of a control group with normal kidney function; (4) study population age >18 years; (5) Asian or Caucasian ethnicity of the populations, and (6) articles providing detailed distribution of ACE genotypes. Studies investigating the relationships between genetic polymorphisms and other kidney diseases (lupus nephritis, polycystic kidney disease, endemic nephropathy, or reflux nephropathy) were excluded.

### Data Extraction and Quality Assessment

Two reviewers (Chin Lin and Sui-Lung Su) independently extracted the data and assessed risk of bias. We recorded the first author’s name, year of publication, ethnicity of the study population, kidney function of the cases, definition of the case group and its population characteristics (mean age, proportion of male subjects, body mass index, diabetes mellitus prevalence, hypertension prevalence, and ACE I/D genotype distribution). Ethnicity of the study population was categorized by study area. Subjects in the Arabian peninsula were classified as Caucasian because Arabs were the main race, and subjects in other regions of Asia (excluding Russia) were classified as Asian. Diabetes mellitus and hypertension were defined by plasma glucose level of >126 mg/dL and systolic blood pressure of >140 mmHg. If the article did not report the prevalence of diabetes mellitus and hypertension or the definition did not match, we assumed a normal distribution of plasma glucose level and systolic blood pressure for calculation.

Estimating moderate effects is difficult in meta-analysis using case–control studies. Researchers prefer that studies provide stratified data or matching data, but previous studies have seldom reported these. Fortunately, the characteristics of case groups may be used to estimate moderate effects under the following two conditions: (1) outcomes were rare events, (2) the major independent variable and moderators were independent events.

For example, when the major independent variable is exposure, with values “yes” or “no,” and the moderator is gender, with values “male” or “female,” the variables *p*_1_, *p*_2_, *p*_3_, and *p*_4_ are the outcome prevalence of women without exposure, men without exposure, women with exposure, and men with exposure. The variable *p*_5_ is the proportion of individuals with exposure in the whole population; *p*_6_ is the proportion of men in the population without exposure; and *p*_7_ is the proportion of men in the population with exposure.

When researchers wish to conduct a case–control study, they must survey the exposure proportion in the case and control groups to estimate the odds ratio (OR). The exposure proportions using a stratified view and ORs are as follows:

Exposure proportion among case women:


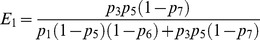


Exposure proportion among case men:


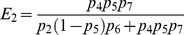


Exposure proportion among control women:


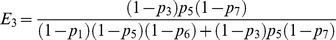


Exposure proportion among control men:


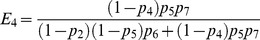


OR in women: 
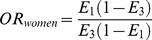


OR in men: 
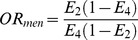


Crude OR (simple combined):


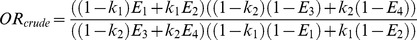
.

*k*_1_ = proportion of males in the case group.

*k*_2_ = proportion of males in the control group.

Two factors (*k*_1_ and *k*_2_) may affect the crude OR. However, when *p*_1_, *p*_2_, *p*_3_, and *p*_4_ are very rare and there is no association between the major independent variable and the moderator (*p*_6_ = *p*_7_),
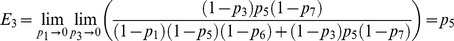


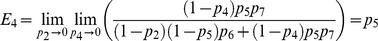


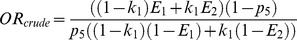



If moderate effects are present (*OR*_women_ ≠ *OR*_men_), the proportion of males in the case group (*k*_1_) is the only factor that can affect the crude OR. In addition, the relationship between *k*_1_ and log of odds ratio approximate the first order polynomial when above assumption were proper. We may accordingly use the characteristics of the case group to estimate the moderate effects in this study under the above assumptions.

Risk of bias was assessed by the following procedures suggested by the Newcastle–Ottawa Quality Assessment Scale [23] (shown in Table S3). This tool assesses studies with a focus on the following factors: (1) selection of study population, (2) comparability between the case and control groups, and (3) the exposure assessed. Each study received a score between 0 and 9. We investigated the relationship between the quality of studies and the estimation of risk.

### Statistical Analysis

Variables are presented as means, proportions, or numbers as appropriate. Our meta-analysis examined the association between ACE I/D polymorphisms and CKD risk for each study by odds ratios (ORs) with 95% confidence intervals (CIs).

The τ^2^ statistic estimated by the DerSimonian–Laird method was used for the assessment of heterogeneity, and a random-effects model based on the Mantel–Haenszel method was applied. Allele type, genotype, and dominant/recessive models were used to calculate the association between genetic polymorphism and CKD risk. This report displays results from the allele type model, unless estimates using a different model were obviously different. Egger’s regression was used to test symmetry of pooled results. Prespecified subgroup analyses included the causes of CKD.

A moderate effect was defined as ratio between ORs in a stratified analysis. For example, if OR for the association between ACE I/D polymorphisms and CKD risk is 6 in the Asian group and 3 in the Caucasian group, the moderate effect of ethnicity will be 6/3 = 2. Possible moderators (ethnicity, age, gender, body mass index, diabetes mellitus, and hypertension) and study characteristic (quality score, study design, and kidney functions of cases) were tested by meta-regression. In multivariable analyses, we adjusted ethnicity because most previous studies have found significant moderate effects [10]–[12]. To assess the effect of ethnicity on gender-dependent effects, we investigated the interaction between gender and ethnicity. The interaction between other moderators and gender were also tested.

This study considered a p-value of <0.05 as significant for all analyses. Statistical analyses were carried out with R, version 2.15.0, using the “metafor” [24] and “meta” [25] packages.

## Results

### Screening Process

**Figure 1:**
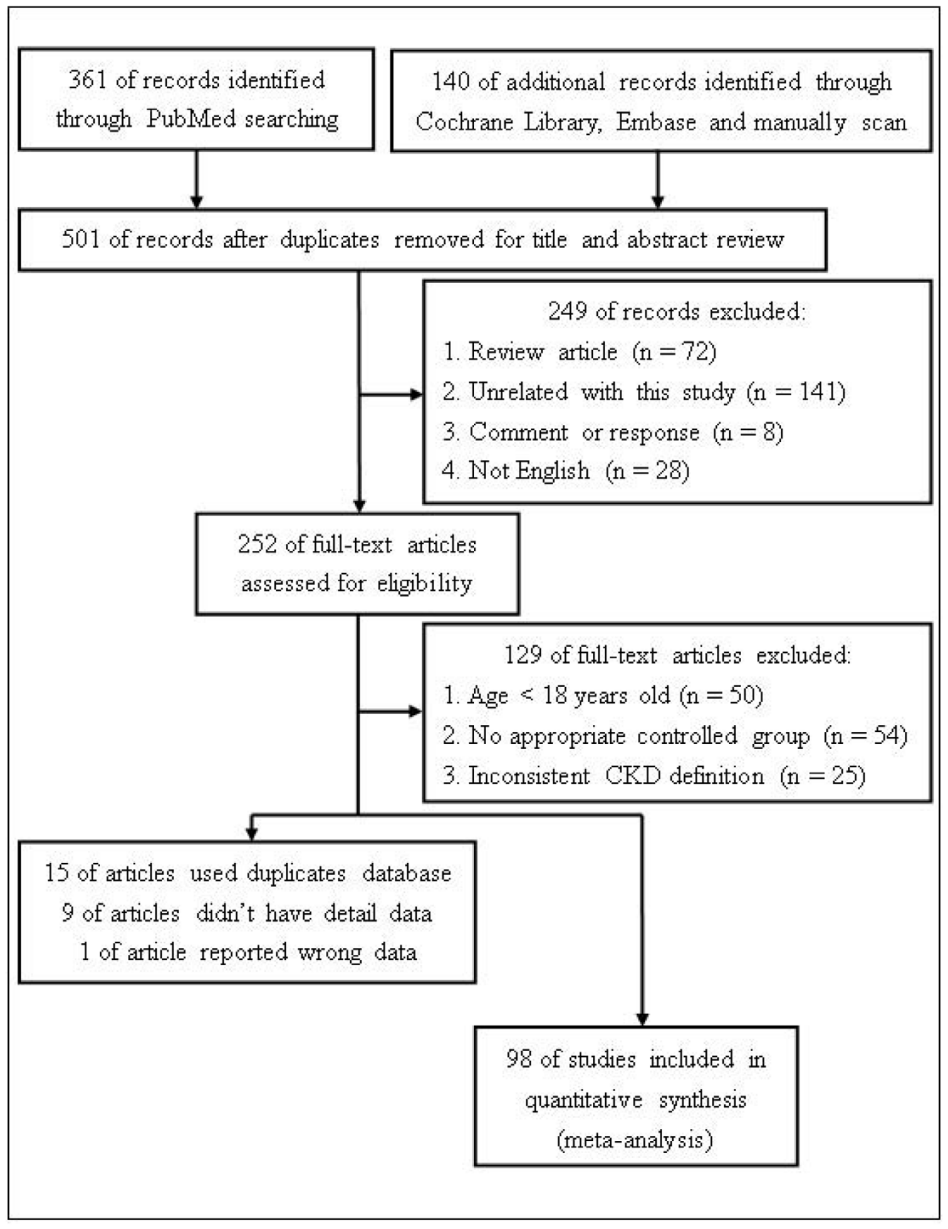
Flow diagram of identification process for eligible studies in this study. n: number of studies were deleted for aforementioned reasons.

Our search strategy returned 501 papers (the identification process is shown in Figure 1). We excluded 249 papers after a preliminary search of titles and abstracts. An additional 129 papers were excluded after full-text articles were assessed, leaving 123 articles that matched our criteria. Of these, 15 used duplicate databases, 9 lacked detailed data, and 1 likely reported wrong data. In 98 of the studies finally included [14]–[19], [26]–[117], 2 (Zsom et al. [30] and Lee et al. [26]) reported results of stratification, but studies by Zsom et al. [30] used a single control group. Accordingly, 99 populations were included in this meta-analysis, and their detailed data are shown in Tables 1 and 2.

**Table 1:** Characteristics of published studies included in this meta-analysis.

First author & year	Ethnicity	Studydesign	CKDtype	Kidneyfunctionof case	Definition of case
Shaikh, 2012 [27]	Caucasian	CC	DN	non-ESRD	UAE >300 mg/day11
Rahimi, 2012 [28]	Caucasian	CS	DN	non-ESRD	ACR >30 mg/g
El-Baz, 2012 [29]	Caucasian	CS	DN	non-ESRD	ACR >30 mg/g
Zsom(1), 2011 [30]	Caucasian	CC	non-DN	non-ESRD	biopsy, ultrasound diagnosed & eGFR <60 ml/min/1.73 m^2^
Zsom(2), 2011 [30]	Caucasian	CC	DN	non-ESRD	proteinuria & eGFR <60 ml/min/1.73 m^2^
Al-Harbi, 2011 [31]	Caucasian	CC	DN	non-ESRD	no description
Jung, 2011 [32]	Asian	CC	GN	non-ESRD	biopsy diagnosed
Ali, 2011 [33]	Asian	CC	Comb	ESRD	doctor diagnosed
Huang, 2010 [34]	Asian	CC	GN	ESRD	biopsy diagnosed
Jayapalan, 2010 [35]	Asian	CS	DN	non-ESRD	ACR >30 mg/g or RRT
Mansoor, 2010 [14]	Caucasian	CS	DN	non-ESRD	albuminuria or RRT
Naresh, 2009 [36]	Asian	CC	DN	non-ESRD	no description
Ezzidi, 2009 [37]	Caucasian	CS	DN	non-ESRD	ACR >30 mg/g or eGFR <90 ml/min/1.73 m^2^
Nikzamir, 2009 [38]	Caucasian	CS	DN	non-ESRD	UAE >30 mg/day
Anbazhagan, 2009 [39]	Asian	CC	Comb	ESRD	RRT
Ahluwalia, 2009 [40]	Asian	CC	DN	non-ESRD	UAE >200 µg/min, ACR >300 mg/g orRRT
Palomo-Piñón, 2009 [16]	Caucasian	CS	DN	non-ESRD	ACR >30 mg/g
Möllsten, 2008 [41]	Caucasian	CS	DN	non-ESRD	ACR >20 mg/g
Eroglu, 2008 [42]	Caucasian	CC	DN	non-ESRD	30 mg/day<UAE <300 mg/day
Arfa, 2008 [43]	Caucasian	CS	DN	non-ESRD	UAE >30 mg/day
Tripathi, 2008 [44]	Asian	CC	non-DN	ESRD	CCr <15 mL/min/1.73 m^2^ & ultrasound diagnosedor CT
Movva, 2007 [45]	Asian	CC	DN	ESRD	SCr >1.5 mg/dL and UAE >30 mg/day
Uddin, 2007 [46]	Asian	CC	DN	ESRD	porteinuria
Buraczynska, 2006 [47]	Caucasian	CC	Comb	ESRD	RRT
So, 2006 [48]	Asian	CS	DN	non-ESRD	ACR >30 mg/g
Shestakova, 2006 [49]	Caucasian	CC	DN	non-ESRD	ACR >300 mg/g
Ng, 2006 [50]	Caucasian	CS	DN	non-ESRD	urinalyses positive, ACR >250 mg/g (men) or >355 mg/g (women)
Prasad, 2006 [51]	Asian	CC	DN	non-ESRD	SCr >3 mg/dL?ACR >200 mg/g
Degirmenci, 2005 [52]	Caucasian	CS	DN	ESRD	UAE >30 mg/day
van der Sman-de Beer, 2005 [53]	Caucasian	CC	Comb	non-ESRD	RRT
Park, 2005 [18]	Asian	CC	DN	ESRD	RRT
Canani, 2005 [54]	Caucasian	CS	DN	non-ESRD	UAE >20 µg/min
Fabris, 2005 [55]	Caucasian	CC	HN	non-ESRD	SCr >1.5 mg/dL
Lau, 2004 [56]	Asian	CC	GN	ESRD	biopsy diagnosed
Suzuki, 2004 [57]	Asian	CC	GN	non-ESRD	biopsy diagnosed
Stratta, 2004 [58]	Caucasian	CC	GN	non-ESRD	biopsy diagnosed
Lochynska, 2003 [59]	Caucasian	CC	GN	non-ESRD	biopsy diagnosed
Papp, 2003 [60]	Caucasian	CC	GN	ESRD	RRT
Aucella, 2003 [61]	Caucasian	CC	Comb	non-ESRD	RRT
Okuno, 2003 [62]	Asian	CS	DN	non-ESRD	UAE >10 µg/min
Ortiz, 2003 [63]	Caucasian	CC	non-DN	non-ESRD	CCr <50 mL/min/1.73 m^2^
Hadjadj, 2003 [15]	Caucasian	CC	DN	non-ESRD	urinary albumin concentration >20 mg/L
Wang, 2003 [64]	Asian	CC	Comb	ESRD	RRT
Dixit, 2002 [65]	Caucasian	CC	GN	ESRD	biopsy diagnosed
Lee(1), 2002 [26]	Asian	CS	non-DN	non-ESRD	UAE >20 µg/min or ACR >20 mg/g
Lee(2), 2002 [26]	Asian	CS	DN	non-ESRD	UAE >20 µg/min or ACR >20 mg/g
Losito, 2002 [66]	Caucasian	CC	Comb	ESRD	RRT
Yoon, 2002 [67]	Asian	CC	GN	non-ESRD	biopsy diagnosed
Fradin, 2002 [68]	Caucasian	CS	DN	non-ESRD	UAE >30 mg/day
Drouet, 2002 [69]	Caucasian	CC	GN	non-ESRD	biopsy diagnosed
Nicod, 2002 [70]	Caucasian	CC	Comb	non-ESRD	RRT
Araz, 2001 [71]	Caucasian	CS	DN	non-ESRD	ACR >30 mg/g
Azar, 2001 [72]	Caucasian	CC	DN	non-ESRD	UAE >30 mg/day
Lovati, 2001 [73]	Caucasian	CC	Comb	ESRD	RRT
Wang, 2001 [74]	Caucasian	CS	Comb	non-ESRD	CCr <60 mL/min/1.73 m^2^
Taniwaki, 2001 [75]	Asian	CS	DN	non-ESRD	UAE >30 mg/day
Viswanathan, 2001 [76]	Asian	CC	DN	non-ESRD	proteinuria >500 mg/dL
Hadjadj, 2001 [77]	Caucasian	CS	DN	non-ESRD	urinary albumin concentration >20 mg/L
Wu, 2000 [78]	Asian	CC	DN	non-ESRD	no description
Hsieh, 2000 [79]	Asian	CC	DN	non-ESRD	proteinuria >500 mg/dL
van Ittersum, 2000 [80]	Caucasian	CS	DN	non-ESRD	UAE >30 mg/day
Tomino, 1999 [19]	Asian	CS	DN	non-ESRD	UAE >20 µg/min or ACR >30 mg/g
Solini, 1999 [81]	Caucasian	CS	DN	non-ESRD	Albuminuria
De Cosmo, 1999 [82]	Caucasian	CC	DN	non-ESRD	UAE >30 mg/day
Miura, 1999 [83]	Asian	CS	DN	non-ESRD	UAE >10 µg/min
Kuramoto, 1999 [84]	Asian	CS	DN	non-ESRD	UAE >15 µg/min
Huang, 1998 [85]	Caucasian	CS	DN	non-ESRD	UAE >30 mg/day
Walder, 1998 [86]	Caucasian	CC	DN	non-ESRD	UAE >30 mg/day
Freire, 1998 [87]	Caucasian	CS	DN	non-ESRD	UAE >30 mg/day
Grzeszczak, 1998 [88]	Caucasian	CS	DN	non-ESRD	ACR >1.9 mg/mol (men) or >2.8 mg/mol (women)
Young, 1998 [89]	Asian	CS	DN	non-ESRD	UAE >30 mg/day
Penno, 1998 [90]	Caucasian	CS	DN	non-ESRD	UAE >20 µg/min
Fernández-Llama, 1998 [91]	Caucasian	CS	HN	non-ESRD	UAE >20 µg/min
Frost, 1998 [92]	Caucasian	CS	DN	non-ESRD	UAE >30 mg/day
Pei, 1997 [93]	Caucasian	CS	GN	non-ESRD	biopsy diagnosed
Marre, 1997 [94]	Caucasian	CS	DN	non-ESRD	UAE >30 mg/day
Barnas, 1997 [95]	Caucasian	CS	DN	non-ESRD	UAE >30 mg/day
Ringel, 1997 [96]	Caucasian	CC	DN	non-ESRD	UAE >30 mg/day
Schmidt, 1997 [97]	Caucasian	CS	DN	non-ESRD	UAE >30 mg/day
Kawada, 1997 [98]	Asian	CC	Comb	ESRD	RRT
Kario, 1997 [99]	Asian	CS	HN	non-ESRD	UAE >15 µg/min
Nakajima, 1996 [17]	Asian	CS	DN	non-ESRD	ACR >30 mg/g
Chowdhury, 1996 [100]	Caucasian	CS	DN	non-ESRD	Albuminuria
McLaughlin, 1996 [101]	Caucasian	CC	Comb	non-ESRD	RRT or doctor diagnosed
Oh, 1996 [102]	Asian	CS	DN	non-ESRD	UAE >20 µg/min
Schmidt, 1996 [103]	Caucasian	CC	Comb	ESRD	RRT
Doi, 1996 [104]	Asian	CS	DN	non-ESRD	ACR >30 mg/g
Ohno, 1996 [105]	Asian	CS	DN	non-ESRD	ACR >10 mg/g
Mizuiri, 1995 [106]	Asian	CC	DN	non-ESRD	UAE >20 µg/min or RRT
Yorioka, 1995 [107]	Asian	CC	GN	non-ESRD	biopsy diagnosed
Fujisawa, 1995 [108]	Asian	CS	DN	non-ESRD	Albuminuria or RRT
Panagiotopoulos, 1995 [109]	Caucasian	CS	DN	non-ESRD	UAE >20 µg/min
Tarnow, 1995 [110]	Caucasian	CC	DN	non-ESRD	UAE >300 mg/day or biopsy diagnosed
Schmidt, 1995 [111]	Caucasian	CC	GN	non-ESRD	biopsy diagnosed
Yoshida, 1995 [112]	Asian	CC	GN	non-ESRD	biopsy diagnosed
Dudley, 1995 [113]	Caucasian	CC	DN	non-ESRD	patients with urine in top tertile of the median UAE
Harden, 1995 [114]	Caucasian	CC	GN	non-ESRD	biopsy diagnosed
Doria, 1994 [115]	Caucasian	CC	DN	non-ESRD	UAE >30 µg/min
Marre, 1994 [116]	Caucasian	CS	DN	non-ESRD	UAE >30 mg/day
Powrie, 1994 [117]	Caucasian	CS	DN	non-ESRD	ACR >3 mg/mmol

CC: case control study; CS: cross-sectional survey; DN: diabetic nephropathy; non-DN: non diabetic nephropathy; GN: glomerulonephritis; HN: hypertensive nephropathy; Comb: combined; ESRD: only ESRD patients; non-ESRD: not only ESRD patients; UAE: urinary albumin excretion rate; ACR: Albumin creatinine ratio; eGFR: estimated glomerular filtration rate; CCr: creatinine clearance; RRT: renal replacement therapy; CT: computed tomography; SCr: serum creatinine.

**Table 2:** Quality score and description of studies’ population in included studies.

First author & year	Quality score	Age (years)	Male (%)	BMI (kg/m^2^)	DM (%)	HT (%)	Case group			Control group		
							II	ID	DD	II	ID	DD
Shaikh, 2012 [27]	7	58	54	27	100	86	18	98	52	123	148	25
Rahimi, 2012 [28]	7	56	40	27	100	63	19	66	55	14	32	26
El-Baz, 2012 [29]	6	59	53		100		4	58	40	5	72	23
Zsom(1), 2011 [30]	4	64	55				39	88	68	44	110	46
Zsom(2), 2011 [30]	4	70	61		100		20	60	34	44	110	46
Al-Harbi, 2011 [31]	4	58	48	30	100	86	12	39	59	25	75	96
Jung, 2011 [32]	7	34	57			61	78	142	41	98	164	38
Ali, 2011 [33]	5	55	55	25	16	41	47	125	18	86	90	14
Huang, 2010 [34]	3	40	50			54	10	29	8	58	52	10
Jayapalan, 2010 [35]	7	60	38		100	42	77	77	21	31	31	19
Mansoor, 2010 [14]	6	53	31	27	100	29	27	45	12	65	102	33
Naresh, 2009 [36]	5	54	53	29	100	95	4	11	15	12	11	7
Ezzidi, 2009 [37]	7	60	46	28	100	50	88	260	167	152	196	54
Nikzamir, 2009 [38]	7	59	56	26	100	54	31	84	64	42	75	28
Anbazhagan, 2009 [39]	7	49	73		28	75	33	58	27	23	53	22
Ahluwalia, 2009 [40]	7	58	66	24	100	59	44	64	132	49	117	89
Palomo-Piñón, 2009 [16]	7	60	48	27	100	26	87	105	43	85	91	24
Möllsten, 2008 [41]	7	47	49		100	52	25	69	27	36	113	48
Eroglu, 2008 [42]	7	58	41	29	100	38	13	17	16	13	24	19
Arfa, 2008 [43]	7	62	43	29	100	68	9	41	40	6	24	21
Tripathi, 2008 [44]	6	36	88		0	69	53	72	55	379	148	42
Movva, 2007 [45]	6	57	70		100		47	88	39	74	74	27
Uddin, 2007 [46]	4	51	54		100	78	12	22	24	24	28	14
Buraczynska, 2006 [47]	8	51	56		19	78	174	346	228	112	268	140
So, 2006 [48]	6				100		407	364	93	549	526	150
Shestakova, 2006 [49]	6	26	48	23	100	26	15	35	13	24	30	12
Ng, 2006 [50]	7	61	61	32	100	48	47	148	96	32	83	52
Prasad, 2006 [51]	7	57	33		100	63	67	74	55	76	97	52
Degirmenci, 2005 [52]	4				100		6	25	12	19	47	30
van der Sman-de Beer, 2005 [53]	6	59	61	26	17	65	110	227	116	112	235	125
Park, 2005 [18]	5	60	58	23	100	83	27	49	27	30	51	7
Canani, 2005 [54]	6				100		66	181	126	120	308	181
Fabris, 2005 [55]	7	60	78	26	0	100	13	32	41	34	83	55
Lau, 2004 [56]	5	43	48				53	48	17	47	43	4
Suzuki, 2004 [57]	5	36	56				42	54	21	111	106	53
Stratta, 2004 [58]	5	50	67			39	23	50	44	29	76	66
Lochynska, 2003 [59]	5	42	74		0	0	13	32	5	20	45	35
Papp, 2003 [60]	4	49	46			65	11	25	14	34	83	33
Aucella, 2003 [61]	5	62	54		16	58	57	201	203	170	576	561
Okuno, 2003 [62]	7	78	50	20	100	31	1	8	3	21	12	5
Ortiz, 2003 [63]	6	56	59	22	0	100	9	71	81	17	66	46
Hadjadj, 2003 [15]	9	66	73	29	100	72	552	1468	1119	115	282	208
Wang, 2003 [64]	5	55	51	23	30	66	106	104	36	71	88	24
Dixit, 2002 [65]	7	24	55				12	26	9	3	24	13
Lee(1), 2002 [26]	8				0		20	45	12	330	277	66
Lee(2), 2002 [26]	8				100		117	137	40	208	170	39
Losito, 2002 [66]	6	67	61	25	14	50	27	81	52	22	84	63
Yoon, 2002 [67]	5	35	56		0	35	44	116	31	70	128	25
Fradin, 2002 [68]	7	57	53	32	100	33	18	61	38	20	54	44
Drouet, 2002 [69]	6	44	77				16	46	63	13	28	36
Nicod, 2002 [70]	5	54	57	25			63	156	41	54	153	53
Araz, 2001 [71]	7	56	41	28	100	63	18	64	34	23	57	43
Azar, 2001 [72]	6	23	46		100		2	27	23	2	7	1
Lovati, 2001 [73]	7	54	57	25	12	51	63	156	41	69	195	63
Wang, 2001 [74]	8						28	99	41	313	662	311
Taniwaki, 2001 [75]	7	61	59	23	100	54	32	40	14	31	26	12
Viswanathan, 2001 [76]	7	57	66	26	100	64	17	45	24	10	8	5
Hadjadj, 2001 [77]	7	40	59	23	100	53	8	34	17	46	116	89
Wu, 2000 [78]	3	60	55		100		22	31	18	19	20	2
Hsieh, 2000 [79]	5	61	49	23	100	60	80	59	40	86	50	21
van Ittersum, 2000 [80]	6	55	59		100	80	23	33	13	53	86	49
Tomino, 1999 [19]	7	61	63		100		312	337	96	163	189	55
Solini, 1999 [81]	8	60	58	30	100	61	33	66	58	27	83	62
De Cosmo, 1999 [82]	7	43	61		100	42	23	76	73	18	53	65
Miura, 1999 [83]	7	36	34		100	21	36	49	13	35	58	10
Kuramoto, 1999 [84]	7	58	61	23	100	56	8	16	9	13	13	3
Huang, 1998 [85]	6				100		4	12	8	7	29	23
Walder, 1998 [86]	7	42	71		100	86	12	25	18	12	16	16
Freire, 1998 [87]	6	28	48		100	17	12	32	33	10	45	34
Grzeszczak, 1998 [88]	7	62		29	100	68	103	230	129	63	118	73
Young, 1998 [89]	7	56	34	25	100	73	23	30	3	26	20	8
Penno, 1998 [90]	5				100		15	38	23	62	258	114
Fernández-Llama, 1998 [91]	5				0	100	1	6	7	5	38	18
Frost, 1998 [92]	5				100		10	7	16	21	48	46
Pei, 1997 [93]	6	49	68				32	81	55	21	49	30
Marre, 1997 [94]	8	43	57	24	100	57	50	168	119	40	69	48
Barnas, 1997 [95]	6	47	70		100	48	9	27	14	15	21	4
Ringel, 1997 [96]	8	51	54	26	100	43	64	152	79	78	199	92
Schmidt, 1997 [97]	7	65	51	29	100	74	61	129	121	62	154	131
Kawada, 1997 [98]	5	59	62		22		89	89	30	84	96	28
Kario, 1997 [99]	6	72	41	25	0	100	42	62	29	86	98	16
Nakajima, 1996 [17]	6	56	64		100		37	50	14	18	19	4
Chowdhury, 1996 [100]	6	39	55		100	97	40	124	78	32	79	55
McLaughlin, 1996 [101]	4	42	57				114	366	312	75	203	93
Oh, 1996 [102]	6	35	42	19	100		12	9	10	11	10	7
Schmidt, 1996 [103]	5	55	58			63	27	51	28	21	38	36
Doi, 1996 [104]	7	62	51	22	100	55	50	85	29	56	56	12
Ohno, 1996 [105]	7	61	53	23	100	56	26	38	15	33	15	9
Mizuiri, 1995 [106]	6	54			100	90	11	51	19	11	11	9
Yorioka, 1995 [107]	5	33	44			6	27	13	8	46	47	10
Fujisawa, 1995 [108]	4				100		24	23	7	17	12	6
Panagiotopoulos, 1995 [109]	5	62	66		100		10	25	15	29	44	42
Tarnow, 1995 [110]	8	41	61	24	100	68	40	95	63	46	77	67
Schmidt, 1995 [111]	6	45	75			59	44	81	79	40	117	77
Yoshida, 1995 [112]	7	39	64			15	20	17	16	19	24	3
Dudley, 1995 [113]	7	53	67	29	100	58	29	82	47	35	87	36
Harden, 1995 [114]	5	35					19	41	40	17	42	39
Doria, 1994 [115]	5	29			100		15	35	24	20	41	16
Marre, 1994 [116]	6	39	60	23	100		4	35	23	15	28	19
Powrie, 1994 [117]	6	35	50	20	100		4	8	7	24	37	24

Quality score: result of quality assessed in each study (detailed data were shown in supplementary file); Age: mean age; Male: probability of male; BMI: mean body mass index; DM: prevalence of diabetes mellitus; HT: prevalence of hypertension; II: number of II genotype carries; ID: number of ID genotype carries; DD: number of DD genotype carries.

### Preliminary Pooled Analyses

**Figure 2:**
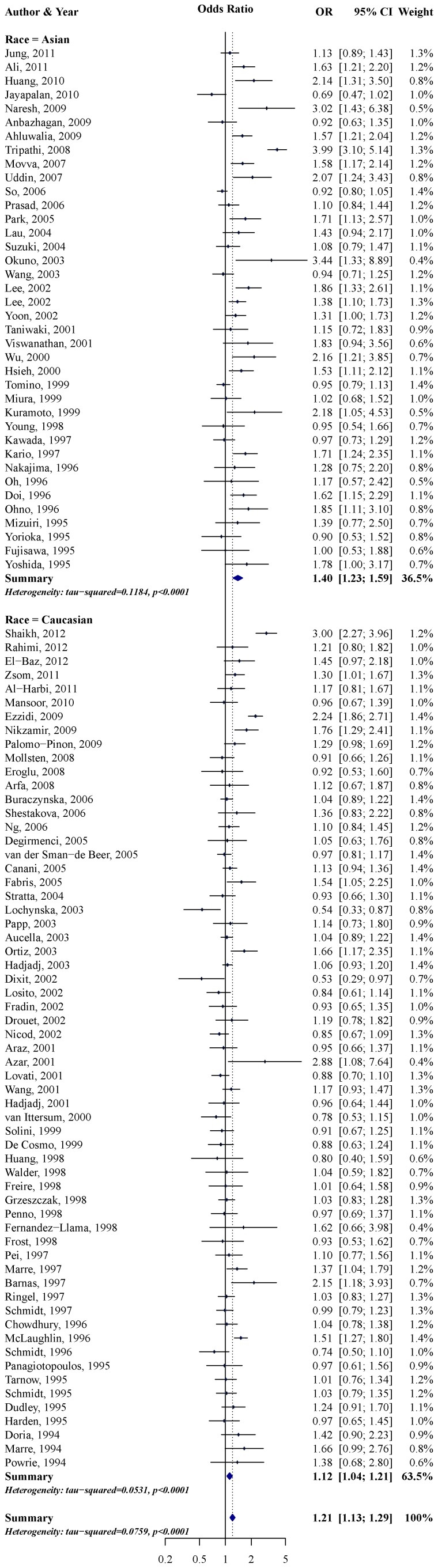
Forest plot of the association between ACE I/D and all-cause CKD using allele type model.

Our meta-analysis showed that a significantly increased CKD risk was associated with the D allele compared with the I allele in each subgroup. Figure 2 shows that D allele carriers had an OR of 1.21 for risk of all-cause CKD compared with I allele carriers, and that these ORs were dissimilar in different ethnicities (*p* = 0.002). OR for CKD in Asian individuals carrying the D allele compared with those carrying the I allele was 1.40 (95% CI: 1.23–1.59), and in Caucasian individuals was 1.12 (95% CI: 1.04–1.21). A summary of the other results is shown in Table 3 and are very similar to those in Figure 2. In the allele type, genotype, and dominant/recessive models, individuals carrying the D allele showed higher CKD risk, and ORs were higher in Asians than in Caucasians. The heterogeneities were higher in Asian populations than in Caucasian populations in each subgroup.

**Table 3:** Odds ratio of ACE I/D and all-cause CKD, diabetic nephropathy, non-diabetic nephropathy using assumption of allele type, genotype, dominant and recessive model.

Model	Ethnicity	All-cause CKD				Diabetic nephropathy				Non-diabetic nephropathy			
		n	OR	95% CI	τ^2^	n	OR	95% CI	τ^2^	n	OR	95% CI	τ^2^
Allele type (D vs. I)	All studies	99	1.21	(1.13, 1.29)	0.076	65	1.23	(1.14, 1.33)	0.063	22	1.29	(1.06, 1.56)	0.166
	Asian	38	1.40	(1.23, 1.59)	0.118	24	1.36	(1.18, 1.56)	0.070	10	1.59	(1.17, 2.17)	0.212
	Caucasian	61	1.12	(1.04, 1.21)	0.053	41	1.17	(1.06, 1.29)	0.064	12	1.08	(0.91, 1.29)	0.054
Genotype-1 (DD vs. II)	All studies	99	1.44	(1.26, 1.64)	0.277	65	1.48	(1.26, 1.74)	0.249	22	1.67	(1.16, 2.41)	0.556
	Asian	38	1.87	(1.46, 2.38)	0.377	24	1.66	(1.27, 2.18)	0.230	10	2.77	(1.62, 4.75)	0.567
	Caucasian	61	1.25	(1.07, 1.46)	0.224	41	1.39	(1.14, 1.71)	0.275	12	1.11	(0.77, 1.60)	0.211
Genotype-2 (ID vs. II)	All studies	99	1.20	(1.10, 1.32)	0.098	65	1.26	(1.13, 1.40)	0.082	22	1.17	(0.92, 1.49)	0.206
	Asian	38	1.34	(1.14, 1.57)	0.152	24	1.31	(1.09, 1.57)	0.088	10	1.43	(0.99, 2.05)	0.259
	Caucasian	61	1.13	(1.02, 1.25)	0.062	41	1.23	(1.07, 1.42)	0.084	12	0.91	(0.74, 1.13)	0.570
Dominant(DD+ID vs. II)	All studies	99	1.28	(1.16, 1.41)	0.132	65	1.33	(1.19, 1.50)	0.110	22	1.30	(1.00, 1.69)	0.271
	Asian	38	1.48	(1.25, 1.74)	0.180	24	1.42	(1.19, 1.70)	0.095	10	1.66	(1.12, 2.45)	0.317
	Caucasian	61	1.17	(1.05, 1.31)	0.100	41	1.28	(1.10, 1.49)	0.130	12	1.01	(0.80, 1.27)	0.031
Recessive(DD vs. ID+II)	All studies	99	1.27	(1.15, 1.39)	0.090	65	1.26	(1.13, 1.42)	0.112	22	1.53	(1.18, 1.99)	0.262
	Asian	38	1.56	(1.28, 1.90)	0.227	24	1.42	(1.12, 1.79)	0.169	10	2.22	(1.42, 3.48)	0.369
	Caucasian	61	1.16	(1.04, 1.28)	0.090	41	1.21	(1.06, 1.37)	0.097	12	1.20	(0.92, 1.57)	0.118

### Identifying Moderators of the Association between ACE I/D Polymorphisms and CKD Risk

Table 4 shows the assessment results of moderate effect on all-cause CKD using the allele type model. Compared with I allele carriers, D allele carriers had a higher OR for CKD risk in Asians than in Caucasians (OR of moderate effect: 1.24; 95% CI: 1.08–1.42). Hypertension also was a moderator (OR of moderate effect: 1.55; 95% CI: 1.04–2.32) and still had a significant moderate effect after adjusting ethnicity (OR of moderate effect: 1.57; 95% CI: 1.07–2.31). No additional moderators were significant after adjustment for ethnicity and hypertension (data not shown). In the final model of these analyses, τ^2^ declined by 15.5% (crude: 0.097; after adjustment: 0.082) and *p* of Egger’s regression test was not significant (*p* = 0.258). We did not find a significant gender-dependent effect.

**Table 4:** Moderator effects of allele type model (D vs. I) on all-cause CKD.

		Unadjusted		Adjusted ethnicity	
	n	OR	95% CI	OR	95% CI
Ethnicity (Caucasian is ref.)	99	1.24*	(1.08, 1.42)		
Study design (CS is ref.)	99	1.05	(0.92, 1.20)	1.03	(0.90, 1.18)
Quality score (per 1 score)	99	0.98	(0.93, 1.04)	0.99	(0.94, 1.05)
Kidney function of case (non-ESRD is ref.)	99	1.03	(0.87, 1.23)	0.97	(0.81, 1.16)
Age (per 10 years)	88	1.02	(0.95, 1.09)	1.01	(0.95, 1.08)
Male (per 100%)	84	1.48	(0.72, 3.01)	1.63	(0.81, 3.28)
BMI (per 5 kg/m^2^)	45	0.86	(0.73, 1.03)	0.95	(0.78, 1.16)
DM (per 100%)	81	0.96	(0.78, 1.18)	0.99	(0.81, 1.22)
Hypertension (per 100%)	68	1.55*	(1.04, 2.32)	1.57*	(1.07, 2.31)

Depend variable: log odds ratio of ACE I/D and all-cause CKD using allele type model.

n: number of studies; OR: odds ratio for moderate effect; 95% CI: 95% confidence interval.

CS: cross-sectional study; non-ESRD: not only ESRD patients; Age: mean age; Male: probability of male; BMI: mean body mass index; DM: prevalence of diabetes mellitus; Hypertension: prevalence of hypertension.

* : p<0.05.

In subgroup analyses of diabetic nephropathy, study design (OR for moderate effect: 1.21; 95% CI: 1.02–1.42) and body mass index (OR of moderate effect: 0.84; 95% CI: 0.70–1.00) had significant moderate effects on ACE I/D polymorphisms and diabetic nephropathy. After adjustment for ethnicity, the moderate effects of study design (OR of moderate effect: 1.19; 95% CI: 1.01–1.41) was still significant but that of body mass index (OR for moderate effect: 0.91; 95% CI: 0.73–1.13) was not (data not shown). The results of the nondiabetic nephropathy subgroup were similar to those for all-cause CKD, with ethnicity (OR of moderate effect: 1.69; 95% CI: 1.07–2.68) and hypertension (OR of moderate effect: 2.42; 95% CI: 1.19–4.93) the only two significant moderators in multivariable analyses (data not shown).

### Estimated Gender-dependent Effects

**Figure 3:**
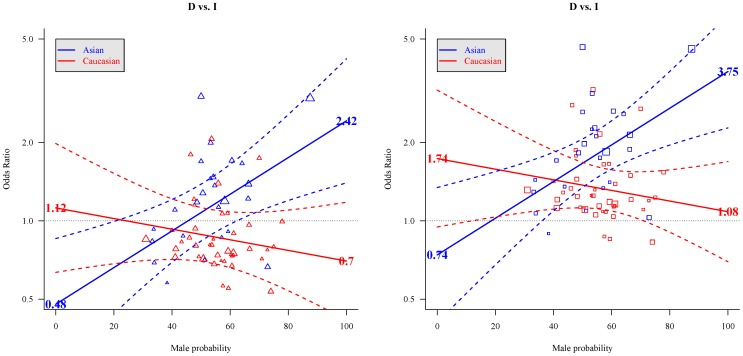
Gender-dependent effects of ACE I/D and all-cause CKD in each population. Left figure was showed the OR in people without hypertension; right figure was showed the odds ratio in patient with hypertension. Red triangle and square: individual studies in Caucasian; blue triangle and square: individual studies in Asian. Solid line: unbiased estimator of odds ratio; dashed line: 95% confidence interval of odds ratios. Estimated value of log odds ratio in individual studies without hypertension used following formula: observed log odds ratio – hypertension prevalence of study population×0.437 (according to Model 2 in Table 5) Estimated value of log odds ratio in individual studies with hypertension used following formula: observed log odds ratio+(1– hypertension prevalence of study population)×0.437 (according to Model 2 in Table 5).

Table 5 shows the interaction of ethnicity and gender. We found no significant interactions between the other variables and gender. Coefficients of interaction were significant for hypertension (*p* before adjustment for hypertension = 0.015; *p* after adjustment for hypertension = 0.003). No other moderators were significant when added to the final model. A proportion of 32.8% of heterogeneity (crude τ^2^ = 0.100; after adjustment, τ^2^ = 0.068) was caused by different ethnicity, gender probability, and prevalence of hypertension in the study population, and *p* of Egger’s regression test was not significant (*p* = 0.217). Figure 3 shows OR of ACE I/D polymorphisms and CKD risk in different combination of ethnicity, gender, and hypertension status based on Model 2 in Table 5. A gender-dependent effect analysis showed the strongest association between the ACE I/D polymorphisms and CKD risk in Asian males with hypertension (OR: 3.75; 95% CI: 1.84–7.65) or without (OR: 2.42; 95% CI: 1.40–4.20).

**Table 5:** Three way interaction of Asian, male and ACE D allele on all-cause CKD, diabetic nephropathy and non-diabetic nephropathy.

	All-cause CKD				Diabetic nephropathy		Non-diabetic nephropathy	
	Model 1		Model 2					
	β	se	β	se	β	se	β	se
**Intercept**	0.297	0.275	0.115	0.291	0.090	0.369	−0.213	0.746
**Race (Caucasian is ref.)**	−0.697	0.391	−0.853*	0.402	−0.817	0.569	−1.080	0.816
**Male (per 100%) €**	−0.312	0.476	−0.470	0.501	−0.122	0.691	−0.349	1.050
**Race×Male**	1.662*	0.686	2.094*	0.715	2.064	1.082	2.666*	1.231
**Hypertension (per 100%) €**			0.437*	0.192	0.229	0.294	0.857*	0.245
**τ^2^**	0.075		0.068		0.081		0.036*	
**Egger’s test**	p = 0.097		p = 0.217		p = 0.385		p = 0.032	

Depend variable: log odds ratio of ACE I/D and CKD using allele type model.

β: coefficients in meta-regression; se: standard error of β.

Model 1: Hypertension was not included in independent variables.

Model 2: Hypertension was included in independent variables.

Egger’s test: p-value of Egger’s regression test.

€: references of parameters were 0%.

* : p<0.05.

Interaction of ethnicity and gender was borderline significant (*p* = 0.056) in the diabetic nephropathy subgroup, but was significant (*p* = 0.030) in the nondiabetic nephropathy subgroup. Although the result of symmetry assessment was significant in nondiabetic nephropathy subgroup (*p* of Egger’s regression test = 0.032), it was noteworthy that 78.3% of heterogeneity (crude τ^2^∶0.166; after adjustment, τ^2^∶0.036) were caused by ethnicity, gender, and hypertension.

**Figure 4:**
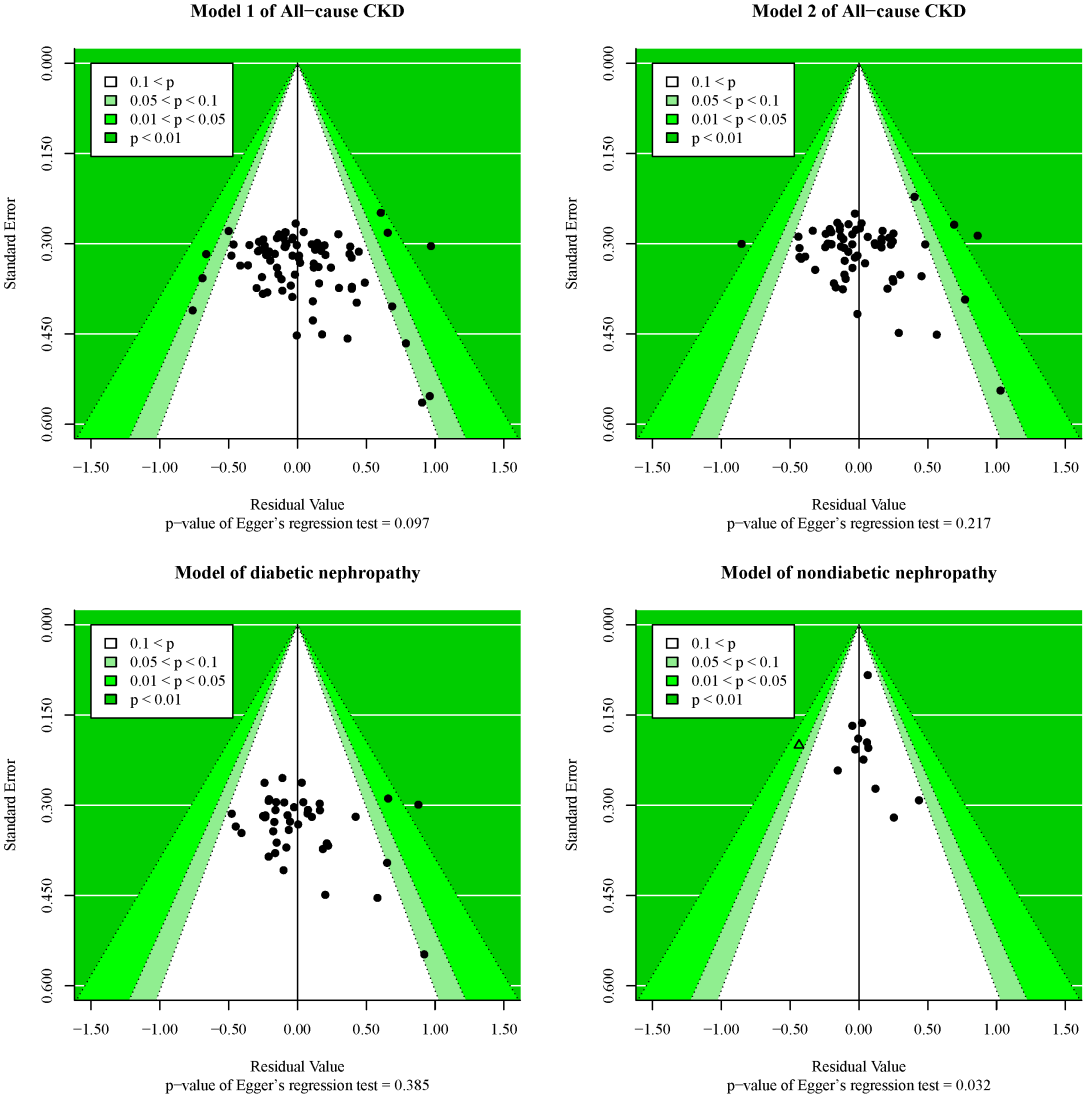
Funnel plot of three way interaction model in each subgroup. The model in nondiabetic nephropathy subgroup was asymmetric. The triangle in that plot was study reported by Jung et[32], and the p value of the student residual was less 0.05. After excluding this study, the p value of Egger’s regression test was not significant (*p* = 0.245) and the moderate effect of interaction and hypertension were more significantly (*p* of interaction: 0.0304→0.0023; *p* of hypertension: 0.0005→<0.0001).

The symmetry of final models was shown in Figure 4. Funnel plots presented the association between residual and standard error based on results of Table 5, and each point represents a study. Egger’s regression test indicated no evidence of publication bias among studies included into the final model this meta-analysis and diabetic nephropathy subgroup. The model in nondiabetic nephropathy subgroup was asymmetric, and it might be due to the study reported by Jung et al. [32]. We did sensitivity analyses leaving the article out (data not shown). The result of symmetry assessment was not significant (*p* of Egger’s regression test = 0.245), and the coefficients in this model were still significant (*p* of interaction effect of ethnicity and gender = 0.002; *p* of moderate effect of hypertension <0.001). In addition, the τ^2^ was 0 in this sensitivity model.

## Discussion

This study showed that CKD risk was higher in D allele carriers than in I allele carriers, and there was no strong evidence that analyses using different model assumptions might produce dissimilar results. Heterogeneity was higher in the Asian population than in the Caucasian population. Interaction between ACE I/D polymorphisms and hypertension exerted an additive effect on CKD risk. A gender-dependent effect of ACE I/D polymorphisms on CKD risk was clearly apparent in Asians but not in Caucasians.

The DD genotype showed higher gene expression and serum ACE levels than the ID genotype, followed by the II genotype [9], [118]. High blood ACE levels may increase blood angiotensin II levels [8], and individuals with higher angiotensin II levels may have a higher CKD risk [119], [120]. Previous studies showed that the association between ACE I/D polymorphisms and CKD risk might not be dominant or recessive [8], [9], [118]. Previous meta-analysis studies showed the supported results, they reported that DD genotype had higher risk of CKD than ID genotype, followed by the II genotype. We also observed the apparent linear association between numbers of D allele and odds ratios compared the II genotype in genotype analyses [10]–[12], [20]–[22]. The assumption of the allele type model in this association might be more reasonable, and it may thus be true that individuals carrying the D allele have a higher CKD risk.

Hypertension in some patients is due to a dysfunction of RAS such as abnormal secretion of renin, causing increased blood angiotensin I levels [121]. D allele carriers had higher ACE levels than I allele carriers [118], leading to more efficient conversion of angiotensin I to angiotensin II, resulting in CKD [119], [120]. The mechanism may be an additive effect of hypertension and the D allele. An additive effect was significant in the nondiabetic group but not in the diabetic nephropathy subgroups. The blood levels of advanced glycation end products (AGE) diabetic patients may be high, possibly causing blood pressure increases [122]. We accordingly hypothesize that the probability of hypertension because of a dysfunction of RAS was higher in the nondiabetic nephropathy subgroup than in the diabetic nephropathy subgroup. Thus, the interaction between ACE I/D polymorphisms and hypertension was significant only in the nondiabetic nephropathy subgroup. This hypothesis may require further studies for confirmation.

We found a significant gender-dependent effect of ACE I/D polymorphisms on CKD risk in Asians. In previous studies in Asians, the ORs of the additive effect on the DD genotype of males were 2.94 and 1.41 in Japanese [17] and Koreans [18], respectively. Another study in Japan also reported a positive additive effect of the DD genotype of males [19]. Studies of Caucasians reported contrary results, with an interaction OR of 0.42 in Pakistan [14]. Another two studies in France [15] and Mexico [16] also showed an additive effect between the DD genotype and female gender but not male gender. Although the interaction tests in these studies were not significant, we could observe dissimilar gender-dependent effect in different ethnicity. Previous studies have also reported a different gender-dependent effect of ACE I/D polymorphisms on blood ACE levels in Asians and Caucasians. In a study conducted in China [13], differences in blood ACE levels between DD genotype and other genotypes among men were significantly greater than those in women. On the other hand, a study conducted in Germany [123] reported the opposite result.

Androgens may play a key role in this additive effect. A study has shown that in intact male rats and ovariectomized female rats that received testosterone for 5 weeks, the androgen may have contributed to the decrease in pressure natriuresis [124]. In an animal study, ACE activity was higher in male mice than in female mice, and this gender difference disappeared after gonadectomy [125]. In previous reports, sensitivity to androgens was stated to be higher in Caucasians than in Asians [126]. Blood androgen levels in Caucasians and Asians showed no significant differences [127], [128]. On the basis of previous studies, we hypothesized that the dissimilar gender-dependent effect of ACE I/D polymorphisms on CKD risk in Caucasians and Asians might be accounted for by dissimilar sensitivity to androgens. The gender difference of male sex hormone utilization was higher in Asians than in Caucasians. Therefore, that the additive effect of the D allele and male gender was also higher in Asians than in Caucasians.

In subgroup analyses, the above additive effect was borderline significant in the diabetic nephropathy subgroup, but there was no evidence that diabetic mellitus might contribute to this additive effect. Although the additive effect also could explain why two populations with different ethnicities had different heterogeneity before adjustment for any moderators, the calculated risk ratio of ACE I/D polymorphisms on CKD risk may have been affected by the gender-dependent effect in Asians.

Our study had three limitations. First, we relied on tabular data rather than on individual patient data, possibly leading to an inflated standard error in pooled analyses. However, we still observed a significant gender-dependent effect difference in different ethnicities. Second, estimates of diabetes mellitus and hypertension prevalence did not factor in the effects of therapy for them. Some subjects having higher blood glucose and blood pressure may have taken drugs, leading to normal biochemical values in reports. Third, we may have missed unpublished data for the nondiabetic nephropathy subgroup. But the results of this subgroup were similar to the results of previous studies and we still observed a significant result excluding the greatest impact of symmetry study; therefore, there is no evidence to question their reliability.

In conclusion, CKD risk was higher with the D allele than with the I allele. Asian ethnicity and hypertension had positive moderate effects, and their effects were more likely to be higher in patients with nondiabetic nephropathy. A gender-dependent effect of ACE I/D polymorphisms on CKD risk was confirmed in Asians; the D allele showed 3.75-fold greater risk for CKD than the I allele in hypertensive Asian males. These results suggest that Asian males should be offered testing for defects in ACE I/D polymorphisms, especially if they are hypertensive. We suggest that physicians should provide specific protection to D-allele carriers, for example by administering ACE inhibitors to hypertensive patients.

## Supporting Information

Table S1 **PRISMA 2009 Checklist.**
(DOC)

Table S2 **Search strategies.** Web sites and uniform resource locator: **MEDLINE**: http://www.ncbi.nlm.nih.gov/pubmed **Cochrane Library**: http://www.thecochranelibrary.com **Embase**: https://www.embase.com
(DOC)

Table S3 **Quality assessment tool in this meta-analysis based on Wells et al.** [**23**] **.**
(DOC)
