# Supplementary material for: Angiotensin-Converting Enzyme Insertion/Deletion Polymorphism Contributes High Risk for Chronic Kidney Disease in Asian Male with Hypertension–A Meta-Regression Analysis of 98 Observational Studies
Source: PLoS One. 2014 Jan 31;9(1):e87604. doi: 10.1371/journal.pone.0087604 (PMC3909221; doi:10.1371/journal.pone.0087604)
Supplement: Table S2 — Search strategies. Web sites and uniform resource locator: MEDLINE: http://www.ncbi.nlm.nih.gov/pubmed Cochrane Library: http://www.thecochranelibrary.com Embase: https://www.embase.com (DOC) [file pone.0087604.s002.doc]

**Table S2** Search strategies.

| **Relevant text of ACE I/D**   1. Peptidyl-Dipeptidase A 2. Angiotensin Converting Enzyme 3. Angiotensin I-Converting Enzyme 4. Antigens, CD143 5. Carboxycathepsin 6. CD143 Antigens 7. Dipeptidyl Peptidase A 8. Kininase A 9. Kininase II 10. ACE 11. I/D 12. insertion/deletion 13. rs4340 14. rs1799752 15. rs13447447 16. rs4646994 17. ((1 or 2 or 3 or 4 or 5 or 6 or 7 or 8 or 9 or 10) and (11 or 12)) or 13 or 14 or 15 or 16   **Relevant text of chronic kidney disease**   1. Renal Insufficiency, Chronic 2. Chronic kidney disease 3. Chronic Kidney Insufficiency 4. Chronic Renal Diseases 5. Chronic Renal Insufficiency | 1. Kidney Insufficiency, Chronic 2. CKD 3. Kidney Failure, Chronic 4. Chronic Kidney Failure 5. End-Stage Kidney Disease 6. End-Stage Renal Disease 7. End-Stage Renal Failure 8. Renal Disease, End-Stage 9. Renal Failure, Chronic 10. Renal Failure, End-Stage 11. ESKD 12. ESRD 13. ESRF 14. Proteinuria 15. Albuminuria 16. Nephropathy 17. Nephritis 18. Dialysis 19. Glomerular filtration rate 20. GFR 21. 24 or 25 or 26 or 27 or 28 or 29 or 30 or 31 or 32 or 33 or 34 or 35 or 36 or 37 or 38 or 39 or 40 or 41 or 42   **Combined (Final strategy)**   1. 17 and 43 |
| --- | --- |

Web sites and uniform resource locator:

**MEDLINE**: <http://www.ncbi.nlm.nih.gov/pubmed>

**Cochrane Library**: [http://www.thecochranelibrary.com](http://www.thecochranelibrary.com/)

**Embase**: https://www.embase.com
