# Supplementary material for: Angiotensin-Converting Enzyme Insertion/Deletion Polymorphism Contributes High Risk for Chronic Kidney Disease in Asian Male with Hypertension–A Meta-Regression Analysis of 98 Observational Studies
Source: PLoS One. 2014 Jan 31;9(1):e87604. doi: 10.1371/journal.pone.0087604 (PMC3909221; doi:10.1371/journal.pone.0087604)
Supplement: Table S3 — Quality assessment tool in this meta-analysis based on Wells et al. [23] . (DOC) [file pone.0087604.s003.doc]

**Table S3 Quality assessment tool in this meta-analysis based on Wells et al. .**

**Selection**

**1) Is the case definition adequate?**

a) Yes, with independent validation

b) Yes, eg record linkage or based on self-reports

c) No description

**2) Representativeness of the cases**

a) Consecutive or obviously representative series of cases

b) Potential for selection biases or not stated

**3) Selection of Controls**

a) Community controls

b) Hospital controls

c) No description

**4) Definition of Controls**

a) No history of disease

b) No description of source

**Comparability**

**1) Comparability of cases and controls on the basis of the design or analysis**

a) Study controls for age and sex

b) Study controls for over than two additional factors

**Exposure**

**1) Ascertainment of exposure**

a) Secure record (eg surgical records)

b) Structured interview where blind to case/control status

c) Interview not blinded to case/control status

d) Written self-report or medical record only

e) No description

**2) Same method of ascertainment for cases and controls**

a) Yes

b) No

**3) Non-Response rate**

a) Same rate for both groups

b) Non respondents described

c) Rate different and no designation
